# Supplementary material for: Developing knowledge-based psychotherapeutic competencies in non-specialist providers: A pre-post study with a nested randomised controlled trial of a coach-supported versus self-guided digital training course for a problem-solving psychological intervention in India
Source: Glob Ment Health (Camb). 2023 Nov 29;10:e87. doi: 10.1017/gmh.2023.81 (PMC10755375; doi:10.1017/gmh.2023.81)
Supplement: Mathur et al. supplementary material [file S205442512300081Xsup001.docx]

**Supplementary Material**

**Table 1: Modules of the Adolescent Problem-solving Training (APT) Course**

| **Number** | **Modules** |
| --- | --- |
| **Basic Counselling Skills** | |
| 1 | Introduction to Adolescent Mental health & Counselling |
| 2 | Counselling Characteristics & Skills |
| 3 | Getting Started |
| 4 | Monitoring Progress |
| 5 | Working Together & Making Shared Decisions |
| 6 | Troubleshooting |
| 7 | Home Practice |
| 8 | Handling Situations of Risk |
| **Problem-Solving Counselling Skills** | |
| 9 | Introduction to Problem Solving |
| 10 | Resource Material |
| 11 | Problem Identification |
| 12 | Option Generation |
| 13 | Option Selection and Making Do-It Plan |
| 14 | Do-it Plan Review |
| 15 | Looking Ahead |
| 16 | Adjusting Delivery |

**Table 2: Final list of competencies**

| **Number** | **Competency** |
| --- | --- |
| 1 | Non-verbal communication |
| 2 | Verbal communication |
| 3 | Explaining confidentiality |
| 4 | Building rapport |
| 5 | Exploring & normalising feelings |
| 6 | Demonstrating empathy, warmth & genuineness |
| 7 | Assessing and managing self-harm |
| 8 | Working collaboratively |
| 9 | Promoting realistic hope for change |
| 10 | Psychoeducation with local terminology |
| 11 | Home practice |
| 12 | Troubleshooting |
| 13 | Identifying target problem |
| 14 | Selecting option(s) |
| 15 | Developing action plan for problem solving |
| 16 | Reviewing action plan |

**Sample Items from KOPS (Knowledge Of Problem Solving) Scale**

Q. What might you do to make Lakshmi feel more at ease when meeting for the first time?

1. Introduce yourself and ask more about Lakshmi’s reasons for coming today.
2. Introduce yourself and share your personal experience of similar difficulties.
3. Introduce yourself and explain the potential outcomes of counselling.
4. Introduce yourself and mention your counselling certifications.

(Competency: Building rapport, Answer: A)

Q. Lakshmi has mentioned her thoughts of not wanting to live anymore. She further requests that you do not inform her parents about these thoughts. How might you respond?

1. Explain to Lakshmi that since she is a minor, you will have to inform her parents.
2. Explain to Lakshmi that discussions from sessions will remain confidential at all times, and you will not inform her parents.
3. Explain to Lakshmi that if you assess her to be at risk, you will first discuss it with her and then inform her parents.
4. Explain to Lakshmi that as long as she promises you that she won’t harm herself you will not inform her parents.

(Competency: Explaining confidentiality, Answer: C)

Q. How might you explain a problem-solving counselling approach to Lakshmi?

1. Counselling will help you to vent about your problems.
2. Counselling will help you to bring positive changes in your personality.
3. Counselling will help you to gain insights into the cause of your distress.
4. Counselling will help you to learn ways to manage your problems.

(Competency: Psychoeducation with local terminology, Answer: D)

Q. Saurabh has not completed his home practice. Which of the following would be a suitable response?

1. Discuss the possibility of Saurabh asking a family member to remind him about doing his home practice.
2. Discuss obstacles to completing the home practice tasks and explore ways of overcoming these.
3. Discuss the downsides of not regularly completing home practice tasks.
4. Discuss whether Saurabh would prefer to practice all tasks in sessions rather than at home.

(Competency: Home Practice, Answer: B)

Q. How might you support Saurabh when it comes to choosing a preferred option for managing his anger?

1. Recommend an option that has worked for other adolescents and ask for his opinion on it.
2. Recommend an option that has a good evidence base from research.
3. Recommend that he gets inputs from his parents and then discusses it with you.
4. Recommend that he lists out the pros and cons of the options that have been generated.

(Competency: Selecting option(s), Answer: D)

Q. What is an important point to keep in mind while helping Saurabh create an action plan for his target problem?

1. The plan should be approved by his teachers and relatives so that they can help him with the potential solution.
2. The plan should be interesting so that he is motivated to implement the potential solution.
3. The plan should be specific about the situations in which he will try out the potential solution.
4. The plan should include a potential solution that has worked for adolescents in the past.

(Competency: Developing action plan for problem solving, Answer: C)
